# A Diversity of Intrinsic Timescales Underlie Neural Computations

**DOI:** 10.3389/fncir.2020.615626

**Published:** 2020-12-21

**Authors:** Sean E. Cavanagh, Laurence T. Hunt, Steven W. Kennerley

**Affiliations:** ^1^Department of Clinical and Movement Neurosciences, University College London, London, United Kingdom; ^2^Wellcome Trust Centre for Neuroimaging, University College London, London, United Kingdom; ^3^Max Planck-UCL Centre for Computational Psychiatry and Aging, University College London, London, United Kingdom; ^4^Department of Psychiatry, Wellcome Centre for Integrative Neuroimaging, University of Oxford, Oxford, United Kingdom

**Keywords:** neuronal timescale, autocorrelation, time constant, decision-making, working memory

## Abstract

Neural processing occurs across a range of temporal scales. To facilitate this, the brain uses fast-changing representations reflecting momentary sensory input alongside more temporally extended representations, which integrate across both short and long temporal windows. The temporal flexibility of these representations allows animals to behave adaptively. Short temporal windows facilitate adaptive responding in dynamic environments, while longer temporal windows promote the gradual integration of information across time. In the cognitive and motor domains, the brain sets overarching goals to be achieved within a long temporal window, which must be broken down into sequences of actions and precise movement control processed across much shorter temporal windows. Previous human neuroimaging studies and large-scale artificial network models have ascribed different processing timescales to different cortical regions, linking this to each region’s position in an anatomical hierarchy determined by patterns of inter-regional connectivity. However, even within cortical regions, there is variability in responses when studied with single-neuron electrophysiology. Here, we review a series of recent electrophysiology experiments that demonstrate the heterogeneity of temporal receptive fields at the level of single neurons within a cortical region. This heterogeneity appears functionally relevant for the computations that neurons perform during decision-making and working memory. We consider anatomical and biophysical mechanisms that may give rise to a heterogeneity of timescales, including recurrent connectivity, cortical layer distribution, and neurotransmitter receptor expression. Finally, we reflect on the computational relevance of each brain region possessing a heterogeneity of neuronal timescales. We argue that this architecture is of particular importance for sensory, motor, and cognitive computations.

## Introduction

Imagine you are listening to Beethoven’s 9th symphony. As you listen, neurons in the auditory cortex are responding to the momentary pitch of the music. In isolation, these momentary pitches are meaningless. The notes must be contextualized across bars (seconds), melodies (tens of seconds), and movements (minutes) for the music to be appreciated and understood. The beauty of the music depends upon melodic expectations that are established over both long and short timescales. The neural processing of information across a diversity of timescales is not only key to many aspects of perception, but also cognition and motor control.

## Timescales of Cortical Regions Reflect Hierarchy

More formally, we will consider the neural temporal receptive field as the length of time over which inputs can be integrated by a neural substrate. Previous work has established the notion of temporal receptive fields, and characterized the temporal properties of neural activity in response to sensory stimuli (Sen et al., 2001; Kiebel et al., 2008; Chen et al., 2015; Hasson et al., 2015). This work has revealed many relevant parallels with the more established concept of spatial receptive fields. It has long been known that neurons in different cortical areas process sensory information across different spatial scales. As information becomes more highly processed, neural representations are based upon larger physical areas and contain more abstract representations which require the integration of multiple sources of sensory information. As a general principle, the size and complexity of spatial receptive fields increase along a visual hierarchy (Lennie, 1998). For example, neurons in the early visual cortex encode the presence of simple features of stimuli (e.g., orientation) in a small, specific area of the visual field (Hubel and Wiesel, 1962, 1968). At the other end of the ventral visual stream, neurons in the inferotemporal cortex encode high-level information about object identity, independent of its location in the visual field (Tanaka, 1996; Brincat and Connor, 2004; Chang and Tsao, 2017). A similar pattern of representational hierarchies is also present in the motor domain, with receptive field sizes increasing and more complex motor representations becoming evident, such as selectivity for sequences of actions, as you move from the primary motor cortex more anteriorly to premotor and prefrontal regions (Luppino et al., 1991; Picard and Strick, 1996; Shima and Tanji, 2000; Nachev et al., 2008; Russo et al., 2020; but see also, Yokoi and Diedrichsen, 2019). Furthermore, in the cognitive domain, complex representations are evident mainly in the prefrontal cortex (Wallis et al., 2001), which also exhibits a hierarchical anatomical organization of abstract representations (Koechlin et al., 2003; Badre, 2008; Nee and D’Esposito, 2016).

When reviewing *temporal* receptive fields, we will initially apply a similar framework and consider how representation size and complexity could vary across neural substrates. In the temporal domain, as a possible equivalent to the neuronal diversity in representing spatial scale, neurons may signal an event (e.g., a sensory stimulus, action, or goal) for varying lengths of time after it occurs. Some neurons may represent this information with a fixed pattern of activity, invariant of how long ago it occurred, within a set temporal window (e.g., 5 s). The length of this window may vary across neurons, and the representation carried by other neurons may be restricted to when the event initially occurs. In higher cortical areas, the temporal receptive window may also be task-dependent, as demonstrated (for example) in working memory tasks with variable delays (Funahashi et al., 1989) or in time estimation tasks with variable durations (Wang et al., 2018). By possessing a spectrum of these representations concurrently, it would allow the brain to hold salient information in working memory while continuing to monitor fluctuations in the environment.

We can also consider the complexity of information in the temporal domain. Stimuli often vary across time, and information must be temporally integrated to enable perception. Further to the musical symphony analogy presented at the start of this piece, another good example is language comprehension (Hasson et al., 2015). To understand speech, the brain integrates auditory information over tens of milliseconds to detect words, which in turn are combined over several seconds to form sentences, which are then integrated across minutes to facilitate the understanding of discourse. Another example would be when we take a journey. The overarching goal of the journey, across many minutes, is to reach a destination. But in order to reach this goal, we set subgoals which are achieved through sequences of actions (across seconds). These action sequences in turn require the precise co-ordination of muscle groups at a timescale of milliseconds. In both of these examples, different neural substrates likely underly the processing of information across different timescales. Therefore, a temporal receptive field may also constitute the length of time over which inputs can be combined or outputs organized—with higher complexity associated with longer integration times.

**Figure 1:**
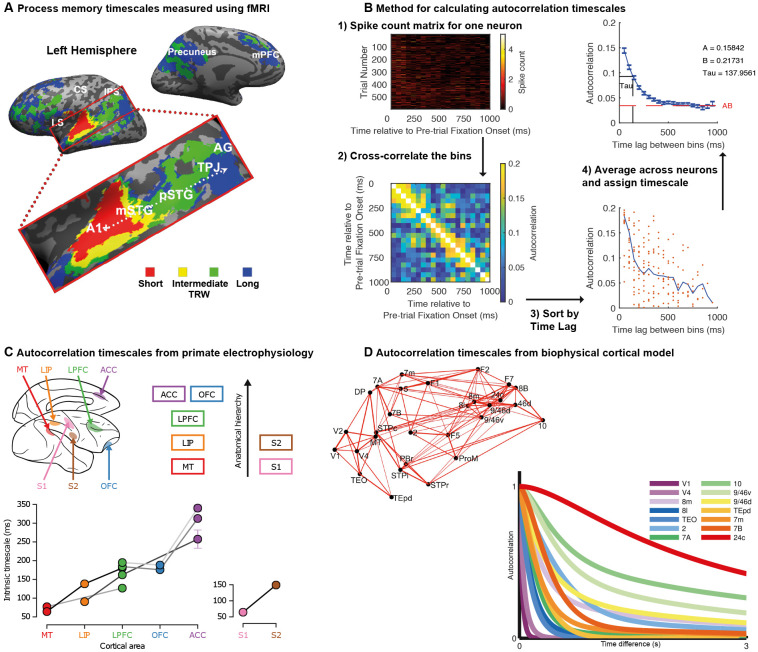
Temporal receptive fields vary across cortical brain regions. **(A)** Topography of temporal receptive fields defined using fMRI data recorded during the passive listening to stories. The color in the voxel heatmap depicts the shortest period to which the original auditory story stimulus could be scrambled and a reliable inter-subject correlation still be obtained (red: story played backward; yellow: a story with word-order scrambled; green: a story with sentence-order scrambled; blue: a story with paragraph-order scrambled). Early auditory areas (A1+) were reliable across subjects even on the most scrambled stimuli, whereas activity in higher regions such as the temporal-parietal junction (TPJ) responded reliably only in the least scrambled condition. There was a gradual hierarchical progression of timescales along the temporal-parietal axis. Data originally published in Lerner et al. (2011); figure reproduced from Hasson et al. (2015); with permission. **(B)** Spike-count autocorrelation method for assigning neuronal timescales. Spike counts for each neuron during the pre-trial fixation periods are subdivided into non-overlapping 50 ms bins. This data from this matrix is correlated across trials to produce a measure of autocorrelation as a function of time-lag between bins. The data is averaged across all neurons recorded in a cortical region before the rate of decay is captured with an exponential fit. The tau parameter determines the intrinsic timescale of the cortical region. **(C)** Intrinsic timescales of seven cortical regions as a function of their position in the anatomical hierarchy. Regions further up in the hierarchy have longer timescales. Each of the different data points (circles) from each brain region were collected by a different research lab, with the lines between datapoints indicating multiple brain areas collected by the same research lab. Reproduced with permission from Murray et al. (2014). **(D)** A large-scale biophysically-realistic neural network simulation shows a hierarchy of timescales in response to visual input. An important feature of the model is the inclusion of anatomical data regarding inter-regional connectivity shown here. In the graph, as in panel **(B)**, autocorrelation is plotted as a function of time lag. Brain regions with more prolonged, stable autocorrelation functions are those at the apex of the cortical hierarchy. Reproduced with permission from Chaudhuri et al. (2015).

Recent work has begun to address how different *cortical regions* process information across different temporal scales. Several studies by Hasson and colleagues have utilized an innovative protocol to demonstrate this with human neuroimaging (see Hasson et al., 2015 for an in depth review; Figure 1A). Human subjects passively experienced a complex stimulus (e.g., listening to a story) across several minutes, before the stimulus was “scrambled” and presented again. For the scrambled versions, the original stimulus was fragmented to different degrees. For instance, some versions only reorganized the paragraphs of the story, whilst others shuffled the order of all of the words. Regardless of the degree of shuffling, fMRI activity recorded in early auditory cortices showed a high degree of inter-subject reliability. However, in higher cortical areas, reliable responses were only observed when scrambled stimuli preserved the structure of paragraphs (Lerner et al., 2011). The interpretation of these results was that early cortical regions processed momentary input regardless of its context, whereas in higher cortical regions information was processed across a much longer timescale. These findings have been demonstrated with various sensory modalities (i.e., auditory, visual, and audio-visual) and with different neuroimaging techniques (Hasson et al., 2008; Lerner et al., 2011; Honey et al., 2012). More recent studies have built on this work to directly infer the timescale over which activity is structured by applying a Hidden Markov Model to the time course of neural activity during movie-watching (Baldassano et al., 2017, 2018). This again reveals a nested hierarchy of timescales from lower to higher cortical areas, with responses in higher areas generalising to an audio description of the same story, while hippocampal activity demarcates high-level boundaries between distinct episodes in the movie. It is notable that a similar hierarchy of timescales can also be found by examining data acquired during the resting state (Stephens et al., 2013), linking these findings to the rich literature on slow timescale interactions between large-scale brain regions while at rest (reviewed in Buckner et al., 2013).

In another line of work, researchers have indexed temporal scales of cortical regions by measuring the spike-count autocorrelation of single neuron activity recorded from macaque monkeys (Ogawa and Komatsu, 2010; Murray et al., 2014). Utilising task independent neural activity recorded during short (~1,000 ms) pre-trial fixation periods, the decay rate of autocorrelation can be captured with an exponential equation and used to define the cortical region’s intrinsic timescale (Figure 1B). When results from a large number of electrophysiological datasets were collated, there was a strong relationship between a region’s position in the anatomical hierarchy (Felleman and Van Essen, 1991; Barbas and Rempel-Clower, 1997) and its intrinsic timescale (Murray et al., 2014; Figure 1C). Moreover, the potential functional relevance of resting spike-count autocorrelation was suggested such that regions with longer intrinsic timescales also contained neurons with longer task-related maintenance of reward information across trials (Bernacchia et al., 2011; Spitmaan et al., 2020).

A large-scale network model of interconnected regions, guided by anatomical data on hierarchical connectivity (Markov et al., 2014a) and local recurrent connectivity (Elston et al., 2011), was sufficient to reproduce this variation in intrinsic timescales (Chaudhuri et al., 2015; Figure 1D). In the model, individual neurons are embedded within densely interconnected networks. Areas of the frontal cortex are densely connected with multiple areas, whereas sensory areas have lower, and typically more local, connection densities (Chaudhuri et al., 2015; Wang and Kennedy, 2016). These connection patterns form cortical hierarchies, defined by asymmetric local (interlaminar) and extrinsic (long-range) connections (Bastos et al., 2015; Chaudhuri et al., 2015; Wang and Kennedy, 2016). These anatomical hierarchies result in long integrative timescales of neurons in frontal cortex, contrasted with short timescales of neurons in sensory areas (Romo et al., 1999; Wang, 2001, 2020; Kiebel et al., 2008; Benucci et al., 2009; Chaudhuri et al., 2015; Wang and Kennedy, 2016).

Although in this initial work variation in intrinsic timescales had only been assigned to brain regions as a whole, perhaps individual neurons within those regions were also capable of processing information across a diversity of timescales. For example, previous research on spatial receptive fields has demonstrated that although there is a general trend of higher cortical regions exhibiting larger spatial receptive fields (Lennie, 1998), in studies where larger numbers of visual neurons were recorded, a significant amount of within-region heterogeneity is also found (Blasdel and Fitzpatrick, 1984; Gur et al., 2005; Nauhaus et al., 2016; Siegle et al., 2019). It was therefore crucial to test whether single neurons had individual timescales, which varied *within* cortical regions.

If single neurons did indeed have their own temporal receptive fields, what would this imply for their roles in cognitive function? It is already established that there is a large degree of heterogeneity with which neurons in higher brain regions are involved in cognitive computations (Shafi et al., 2007; Jun et al., 2010; Wallis and Kennerley, 2010; Meister et al., 2013). It might therefore be the case that a neuron’s intrinsic timescale determines its functional role in extended cognitive processes, such as decision-making and working-memory—specifically the neuron’s strength and dynamics of information encoding. This could be examined by relating an individual neuron’s encoding properties with its own intrinsic timescale, as opposed to the broader timescale of the brain region it inhabited.

Several studies have begun to address these questions with single neuron electrophysiology experiments in macaque monkeys. Here, we will review this work and consider its significant implications—specifically what it may tell us about how neural circuits are organized, how they compute information, and how we should go about studying them in future.

## A Diversity of Timescales at The Single Neuron Level

**Figure 2:**
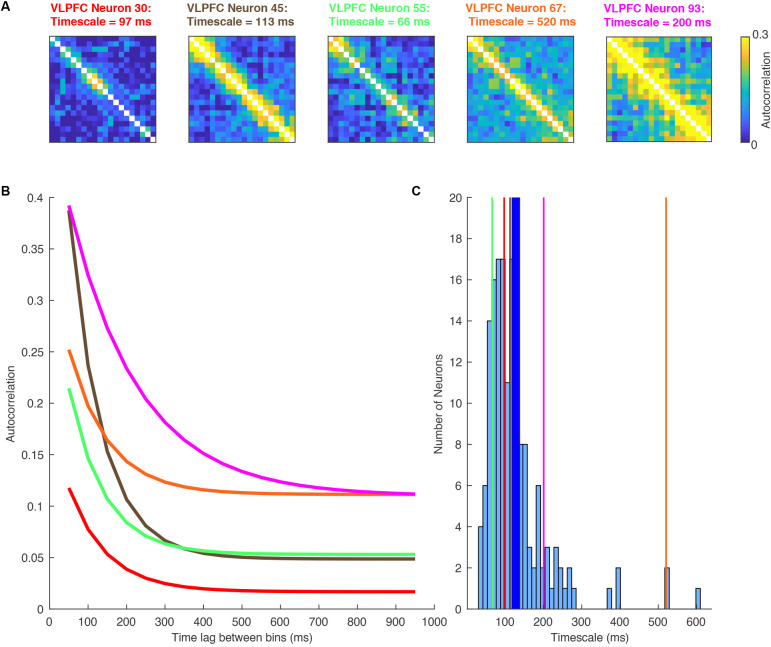
A Heterogeneity of single-neuron timescales exist within a brain region. Data recorded from the ventrolateral prefrontal cortex (VLPFC) during a working memory task (Cavanagh et al., 2018). **(A)** Autocorrelation structure of five VLPFC neurons, plotted as a function of time within the pre-trial fixation period. As in Figure 1B, these are calculated by correlating the spike count autocorrelation across trials. Despite being recorded in the same brain region, there is a large degree of diversity. **(B)** Autocorrelation structure of VLPFC neurons, plotted as a function of the time lag between bins. As in Figure 1B, the data from above have been sorted by the time lag. Each of the lines corresponds to an exponential fit of the decaying autocorrelation of one of the neurons’ heatmaps above (corresponding color). There is substantial heterogeneity in the individual neurons making up the whole region average. Each neuron has an exponential decay reasonably distinct from the population average. **(C)** Histogram showing the single neuron exponential decay time constant assigned to all neurons within VLPFC. The vertical lines mark the example neurons shown in this figure. The thicker blue line marks the population mean.

**Figure 3:**
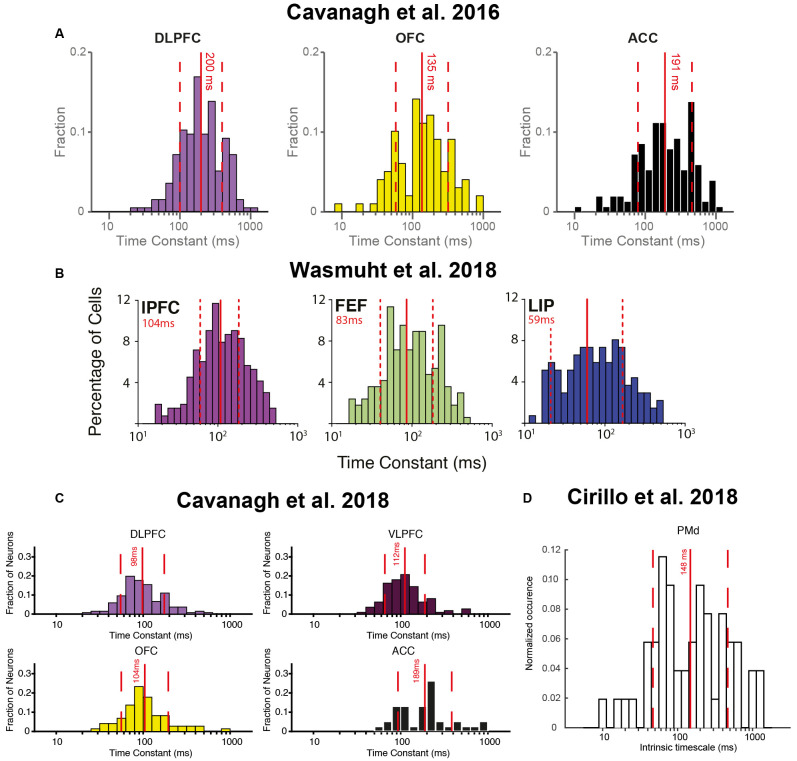
Heterogeneity of single-neuron timescales exist within multiple brain regions across several studies. **(A)** Histograms of single-neuron timescales for dorsolateral prefrontal cortex (DLPFC), orbitofrontal cortex (OFC), and anterior cingulate cortex (ACC). Data were recorded during a value-based decision-making task. Adapted from Cavanagh et al. (2016), where originally published with a CCBY4 licence. **(B)** Histograms of single-neuron timescales for lateral prefrontal cortex (lPFC), frontal eye field (FEF), and lateral intraparietal area (LIP). Data were recorded during a change-detection working memory task. Adapted from Wasmuht et al. (2018), where originally published with a CCBY4 licence. **(C)** Histogram of single-neuron timescales for DLPFC, VLPFC, OFC, and ACC. Data were recorded during an oculomotor delayed working memory task. Adapted from Cavanagh et al. (2018), where originally published with a CCBY4 licence. **(D)** Histogram of single-neuron timescales for dorsal premotor cortex (PMd), recorded during a rule-based working memory task. Adapted from Cirillo et al. (2018), where originally published with a CCBY4 licence.

One of the first studies to examine single neuron intrinsic timescales using spike-count autocorrelation was (Cavanagh et al., 2016), which utilized electrophysiology data recorded from macaque monkeys during a value-based decision-making task (Hosokawa et al., 2013). Before the monkey began to make a choice, there was a 1,000 ms fixation period on each trial. The same spike-count autocorrelation analysis was applied (Murray et al., 2014; Figure 1B), with one important difference. Instead of pooling the autocorrelograms of all neurons within a brain region, a timescale was fitted for each individual neuron. Although this inevitably made the fitting process more noisy, and some neurons were poorly described by a simple exponential decay, the majority of neurons exhibited a decay in autocorrelation structure reliably quantified by an exponential function (see Figure 2 for examples). Importantly, this analysis highlighted a striking degree of *within-region* variability (Figure 2)—even within the anterior cingulate cortex (ACC), which sat at the apex of the hierarchy identified in Murray et al. (2014); there was a spectrum of timescales including many neurons with short timescales. Further studies then applied the same single-neuron analysis to many different brain regions throughout the cortical hierarchy (Figure 3), including posterior parietal cortex (Wasmuht et al., 2018), lateral prefrontal cortex (lPFC; Cavanagh et al., 2016, 2018; Wasmuht et al., 2018; Fascianelli et al., 2019; Fontanier et al., 2020; Kim and Sejnowski, 2020), orbitofrontal cortex (OFC; Cavanagh et al., 2016, 2018; Fascianelli et al., 2019), cingulate cortex (Cavanagh et al., 2016, 2018; Fontanier et al., 2020), premotor cortex (Cirillo et al., 2018), and frontopolar cortex (Fascianelli et al., 2019). All of these regions contained single neurons with a diversity of timescales, suggesting that brain regions possessing a heterogonous distribution of timescales is a generalized feature of cortical organization. Despite this work predominantly focussing on higher-level cortical regions, it would be reasonable to predict that lower-level sensory regions may also contain heterogenous timescales. This appears likely from single-neuron autocorrelograms (Murray et al., 2014), and is supported by a previous study which observed heterogeneity of autocorrelation decay when recording intracellularly from neurons in cat striate cortex (Azouz and Gray, 1999). Variability in single-neuron timescales has also been observed in several early visual areas of the mouse brain (Siegle et al., 2019), and it will be interesting to explore this more conclusively in future studies of primate cortex.

**Figure 4:**
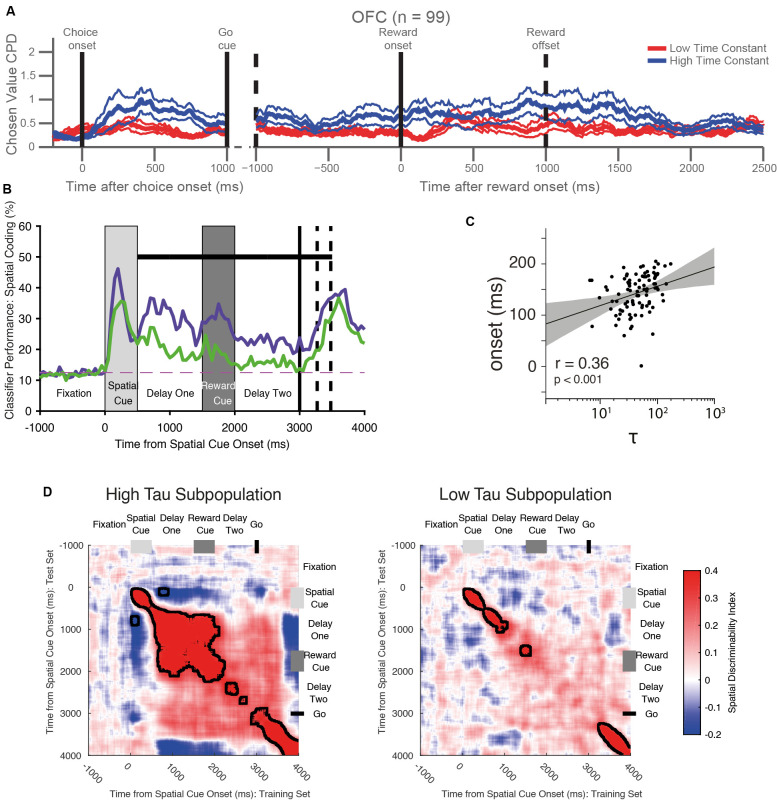
Functional roles of single-neuron timescales during cognitive tasks. **(A)** Long timescale neurons in the orbitofrontal cortex (OFC) are more involved in decision-making and the maintenance of value information until the outcome. The graph shows the coefficient of partial determination (CPD) of chosen value coding for long timescale (blue) and short timescale (red) neurons within OFC. Long timescale neurons have stronger value coding at the time of choice, then throughout the trial until the end of the outcome period. Adapted from Cavanagh et al. (2016) where originally published with a CCBY4 licence. **(B)** Long timescale neurons in the ventrolateral prefrontal cortex (VLPFC) are more involved in the maintenance of spatial working memory information. The graph shows the accuracy with which a linear classifier could decode the remembered spatial location from a subpopulation of neurons with long (purple) and short (timescales). The neural population with longer timescales shows stronger signaling of working memory information—specifically during the delay period. The dashed horizontal line shows chance-level classifier performance. The black horizontal bar shows a significant difference between the two populations. Adapted from Cavanagh et al. (2018) where originally published with a CCBY4 licence. **(C)** Correlation between the onset latency of significant stimulus encoding with the intrinsic timescale in the lateral prefrontal cortex. There is a significant correlation—neurons with shorter timescales encode information more quickly following stimulus onset. Each dot represents one neuron, the black line indicates a linear fit to the data with the shaded area depicting the 95% confidence interval of the fit. Adapted from Wasmuht et al. (2018) where originally published with a CCBY4 licence. **(D)** VLPFC long-timescale neurons have more stable working memory encoding than VLPFC short timescale neurons. The heatmaps show the cross-temporal stability of spatial coding in the two populations. In the long timescale subpopulation, there is greater stability of spatial coding: the off-diagonal elements are warm in color, meaning that the same population code persists throughout the delay epoch following the spatial cue. Although a stable state is reached during delay-one, this is disrupted by the presentation of the distracting reward cue, and there is only a weak non-significant cross-temporal generalization between delay-one and delay-two. In the low time-constant population, coding is always dynamic (i.e., on diagonal heat), so no stable state is established. Adapted from Cavanagh et al. (2018) where originally published with a CCBY4 licence.

Once it had been established single neurons possessed different individual timescales, these studies next investigated whether this variation was functionally significant. A simple way to approach this question was to test the relationship between timescales quantified during the resting (fixation) period of the task with the strength of a neuron’s subsequent task-related activity. For example, encoding of the value of the chosen option during a decision making task may arise as a consequence of the process of evidence integration during a temporally extended decision process (Hunt et al., 2012, 2015), or may also support maintaining value information until later in the trial when learning can occur by assigning credit to the chosen option (Rangel and Hare, 2010; Jocham et al., 2016; Enel et al., 2020). Because both evidence integration and working memory for value are temporally extended processes, it might be expected that single neurons with longer intrinsic timescales are more involved in these cognitive processes. This relationship could be explored by correlating timescales with the coding strength of each neuron, or alternatively by subdividing a brain region’s entire population by a median split of timescales and comparing the task-related activity in the two groups (Cavanagh et al., 2016). Together, these analyses revealed that prefrontal neurons with longer timescales exhibited stronger chosen value coding when a decision was being made. Moreover, long timescale neurons in OFC continued to signal the chosen value until an outcome was received (Figure 4A). Neurons with longer timescales were therefore more involved in both choice and maintaining a representation of the expected outcome across delays which could support credit assignment processes.

The apparent relationship between intrinsic timescales and task-related processing can also be extended to another cognitive process—working memory. Whereas decision-making requires the gradual integration of evidence across time (Gold and Shadlen, 2007), working memory involves the maintenance of task-relevant information in the absence of direct sensory input (Goldman-Rakic, 1995). A number of studies demonstrated that neuronal timescales predicted the strength of mnemonic encoding on a variety of different working-memory paradigms, with longer timescale neurons again playing a greater role in the maintenance of mnemonic information (Nishida et al., 2014; Cavanagh et al., 2018; Cirillo et al., 2018; Wasmuht et al., 2018; Fascianelli et al., 2019; Fontanier et al., 2020; Kim and Sejnowski, 2020; Figure 4B). This effect was present in multiple brain regions [lPFC, cingulate cortex, frontal eye field (FEF), premotor cortex], and for multiple different modalities of mnemonic information (spatial location/response direction, expected reward size, stimulus color).

While the results discussed so far had uncovered the relationship between neuronal timescales and the *strength* of encoding, they did not address another important computational property: the *pattern* with which this information was encoded. The temporal dynamics of population encoding has become of increasing interest and controversy in both decision-making and working memory research fields (Latimer et al., 2015; Constantinidis et al., 2018; Lundqvist et al., 2018). Competing explanations propose that the pattern of neural encoding is either stable (Constantinidis et al., 2018), time-varying (Lundqvist et al., 2018) or even activity-silent (Stokes, 2015), during working memory. While these discrepancies may relate to the task paradigm studied, or the brain region recorded from, it also possibly reflects the inherent neuronal properties—such as intrinsic timescales—of the cells sampled. To explore this, two studies compared the cross-temporal encoding dynamics of short and long timescale neurons (Cavanagh et al., 2018; Wasmuht et al., 2018). When the population of lateral prefrontal neurons were split according to their timescale, the group with longer timescales exhibited stable mnemonic coding whereas those with shorter timescales displayed dynamic coding (Figure 4D). These results reveal that in addition to the strength of encoding, intrinsic timescales can also explain computational dynamics, which here has proven useful in reconciling stable and time-varying working memory theories. While in these two studies the target of working memory was an object or spatial array, a similar separation of stable and dynamic subspaces has recently been found for value-coding neurons in OFC and ACC as well, suggesting this may be a general property of PFC coding (Enel et al., 2020).

Surprisingly, although long timescale neurons exhibited a stable pattern of encoding, this was disrupted by the presentation of a salient distractor (Cavanagh et al., 2018; Figure 4D). One may have predicted that the maintenance of stable encoding would be important to shield mnemonic information from distraction, and that long timescale neurons would be essential for this process. This result may instead indicate that the function of these long timescale neurons is the *integration* of multiple pieces of task-relevant information, rather than the stable *maintenance* of individual pieces of information. These alternative hypotheses tie directly in to the ideas proposed at the start of this review: is the function of a temporal receptive field to maintain information for a fixed time window, or to integrate all of the information occurring within that window? Unfortunately, all of the cognitive paradigms reviewed so far have been unable to arbitrate between these two hypotheses because the task-relevant stimuli do not vary sufficiently across time. Although the decision-making task discussed earlier (Cavanagh et al., 2016) involves the gradual integration of implicit, noisy value estimates across time (Gold and Shadlen, 2007; Hunt and Hayden, 2017), these internal estimates are not accessible to the experimenter. Future work could utilize a decision-making paradigm with experimenter controlled time-varying evidence (Kira et al., 2015; Cavanagh et al., 2020), which requires the combination of many different stimuli. A paradigm such as this dissociates individual information from the integrated total, and would help to determine whether intrinsic timescales better predict a functional role in information integration or maintenance.

So far, most of the research in this area has focussed on how long timescale neurons may be more functionally important for extended cognitive processes. However, there has been less evidence presented regarding the possible roles of short timescale neurons. This has been addressed by a recent study which demonstrated that during an inter-trial period neurons with short timescales encoded momentary feedback information more strongly (Fontanier et al., 2020). This contrasted with long timescale neurons, which at this point of the task preferentially encoded information which was relevant for future decisions which would occur in subsequent trials. Additionally, there has also been some evidence to suggest that neurons with shorter timescales may encode information at a shorter latency (Wasmuht et al., 2018; Figure 4C). It is unknown whether neuronal timescale varies as a function of cortical layer, but as we discuss further below, this result would be consistent with shorter timescale neurons residing in layer IV (and so receiving earlier input), and longer timescale neurons residing in layers II and III (where local recurrent excitation would allow temporally extended computation to occur). Furthermore, it has been suggested that short timescale neurons may utilize a time-varying dynamic representation in order to increase coding dimensionality (Wasmuht et al., 2018)—a computational feature which may be crucial for complex behavior (Rigotti et al., 2013). However, these ideas will have to explored more specifically in future studies (see also section on “Computational Advantages of a Diversity of Within-Region Neuronal Timescales”).

In addition to quantifying the rate of exponential decay of spike-count autocorrelation, it is important to consider other features of the autocorrelograms. Single-neuron autocorrelograms also significantly vary in their offset, and the importance of this parameter has yet to be explored. One recent study also identified important heterogeneity in the initial time-lag before autocorrelation begins to decay as a function of time (Fontanier et al., 2020), a feature which was particularly prominent in cingulate cortex (Murray et al., 2014; Cavanagh et al., 2016, 2018; Fontanier et al., 2020). Related to the time-lag of autocorrelograms, other studies in rodents have demonstrated diversity in the time-lag of stimulus representations (Harvey et al., 2012; Morcos and Harvey, 2016; Scott et al., 2017). This pattern of activity could arise from network architectures facilitating the sequential activation of individual neurons, and may be a mechanism through which a dynamic population code could underlie the retention of information in working memory (Goldman, 2009; Rajan et al., 2016).

Aside from analyzing resting spike-count autocorrelation, other researchers have devised different methods to quantify single neuron temporal receptive fields (Bernacchia et al., 2011; Scott et al., 2017; Dragomir et al., 2020; Hart and Huk, 2020; Spitmaan et al., 2020). The majority of these have focussed on the temporal dynamics of task-related encoding—highlighting a heterogeneity for the duration of information maintenance across neurons within the same brain region. An advantage of the autocorrelation approach is that by considering resting activity, it can quantify the intrinsic properties of the neuron, and then determine how these intrinsic properties influence the neuron’s role in computations. Hence, this approach can provide broader insights about the underlying cortical architecture (see also later section on “The Anatomical and Biophysical Basis of Single Neuron Timescales”). Furthermore, during the pre-trial fixation period, the subjects are in a controlled, attentive state without eye movements or knowledge of the forthcoming task stimuli. This minimizes the potential confounds of any task-related responses, and facilitates an analysis method that can be applied and compared across many datasets. However, there are also important advantages to quantifying timescales using patterns of task selectivity—such as having access to a greater amount of data than that limited to the fixation period. This may help to identify neurons with timescales much longer than can be captured with an exponential limited to a 1,000 ms fixation window. A further advantage of this method is being able to relate the extracted timescales more directly to behavior. These timescales may be far longer than those quantified using resting autocorrelation (Bernacchia et al., 2011; Spitmaan et al., 2020), and the two may or may not be directly related (Spitmaan et al., 2020). While quantifying timescales directly using task selectivity has provided interesting results, a more detailed discussion of these is outside the scope of this review.

## The Anatomical and Biophysical Basis of Single Neuron Timescales

**Figure 5:**
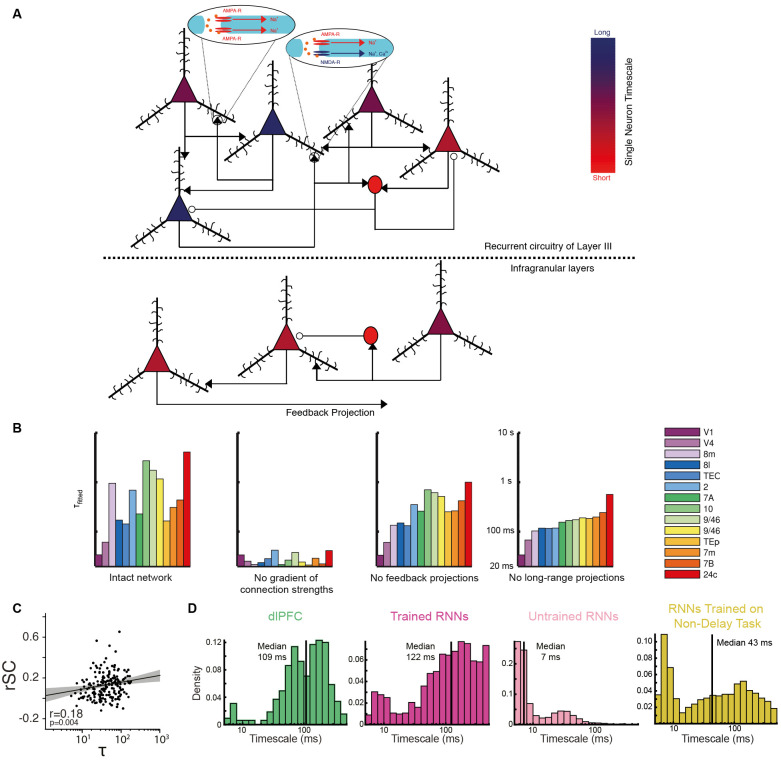
Anatomical and biophysical basis of neuronal timescales. **(A)** Schematic illustration of the circuit properties hypothesized to determine neuronal timescales. The putative timescale of each example neuron is indicated by the color of their cell bodies—with a diverse range spanning from short timescales in bright red to long timescales in dark blue (see also color bar). Excitatory pyramidal neurons have triangular cell bodies and contribute to glutamatergic synapses indicated by black arrowheads. These synapses may contain different relative proportions of AMPA and NMDA receptors [depicted here as a synapse with only AMPA receptors (left oval inset), or a 1:1 AMPA:NMDA receptor ratio at a different synapse (right oval inset) for simplicity]. Inhibitory interneurons have circular cell bodies and contribute to GABAergic synapses indicated by open circles. Above the dashed line is a model circuit of layer III in the primate prefrontal cortex, below the dashed line is a model circuit of an infragranular layer. Several anatomical features are proposed to determine a neuron’s timescale. First, we propose that neurons in layer III generally have longer timescales than those in the infragranular layers. Second, we propose that the longest timescales are observed in excitatory neurons with a greater number of recurrent connections and expressing a higher proportion of NMDA receptors relative to AMPA receptors, and with a greater proportion of NR2B subunits. The shortest timescales are found in the inhibitory interneurons, although more realistic models would include a diversity of interneuron subtypes with potentially differing intrinsic time constants. **(B)** Both local and long-range projections are important in determining the timescales of cortical regions in a biophysical network model of the primate cortex. The histograms show the autocorrelation timescales ($T_Fitted$) calculated from the activity in the intact network (far left), then for various perturbations. When the gradient of increasing numbers of intra-region excitatory synapses further up the cortical hierarchy is removed (center left), the diversity of neuronal timescales is lost entirely. When feedback (center right) and all long-range projections (far right) are removed, there is a reduction in timescale diversity from the intact network. Reproduced with permission from Chaudhuri et al. (2015). **(C)** Evidence that local recurrent connection strengths determine single neuronal timescales. The spike count noise correlation with simultaneously recorded neurons (rSC), an estimate of local connectivity, is positively correlated with neuronal timescale (τ). Each dot depicts a cell recorded from either the lateral prefrontal cortex, the FEF, or the lateral intraparietal area of the macaque cortex. The black line and shaded region denote a regression line with a 95% confidence interval. Adapted from Wasmuht et al. (2018) where originally published with a CCBY4 licence. **(D)** Evidence of single-neuron timescale heterogeneity in trained recurrent neural networks (RNNs). Histograms show the single neuronal timescales from an experimental dataset recorded from the DLPFC (Constantinidis et al., 2016), an RNN trained to perform a delayed match to sample task, an untrained RNN, and an RNN trained to perform a similar choice task without a working memory delay. The RNN trained to perform the temporally extended cognitive process exhibits a diversity of single unit timescales most in keeping with the experimental data. Reproduced from Kim and Sejnowski (2020).

This section will set out to address what factors contribute to the diversity of single neuron timescales (Figure 5A). As a starting point, it will consider factors which have already been suggested to contribute to the diversity of timescales at the level of cortical regions, and try to apply these at a more local level.

### Local Connection Patterns

When addressing the biophysical basis of intrinsic timescales at the single neuron level, it is helpful to first consider existing work probing the determinants of timescales at the level of cortical regions (Chaudhuri et al., 2015). Chaudhuri et al. (2015) developed a large-scale dynamical model of macaque neocortex where each brain area is described by a recurrent network (Figure 1D). Both local and inter-regional circuit mechanisms contributed to a hierarchy of timescales across cortical areas (Figure 5B), which closely resembled the experimental timescales derived from autocorrelation (Murray et al., 2014). A particularly important feature of the model was that regions higher in the cortical hierarchy were endowed with stronger local excitatory connection strength, motivated by the empirical observation that pyramidal neurons in these regions possess a greater number of dendritic spines (Elston, 2000, 2003). However, the experimental evidence suggests there is widespread heterogeneity in spine density within cortical regions (Elston, 2003)—mirroring the variability in single neuron timescales presented in this review. It is therefore important to consider whether local, within region, differences in excitatory connection strength contribute to single neuronal timescales.

By extending the inferences made at the level of cortical regions (Chaudhuri et al., 2015), it is likely that neurons with the longest timescales have the strongest levels of local recurrent connections. One way to examine this hypothesis is to consider noise correlations—the spike count correlation between pairs of simultaneously recorded neurons—as an indirect measure of connection strengths (Cohen and Kohn, 2011). Intriguingly, initial analyses suggest longer timescale cells exhibit higher noise correlations—and hence stronger local connection strengths (Wasmuht et al., 2018; Figure 5C). There is also evidence suggesting that the stable population codes generally observed in higher cortical areas are supported by stronger coupling between neurons (Runyan et al., 2017). In addition to the strength of local connectivity, the architecture of those connections may be of relevance. Aside from the temporal domain, it has been shown directly that neurons in mouse primary visual cortex with stronger connectivity share more similar spatial receptive fields (Cossell et al., 2015; Lee et al., 2016). Future studies could examine whether there is a similar association for temporal receptive fields. Interestingly, theoretical work has proposed classes of network architecture that could facilitate a diversity of timescales differentially concentrated in separate parts of the wider network. This can be realised through localized eigenvectors in the network’s connectivity matrix (Chaudhuri et al., 2014).

Significant insights into the role of local connectivity in single neuron timescales have also been provided by computational modeling. Computational accounts have stressed the importance of heterogeneous local connection weights for producing a diversity of single neuron timescales (Bernacchia et al., 2011; Chaudhuri et al., 2014). A separate body of theoretical work has also investigated how a closely related temporal feature of neural activity, population sequences where individual neurons have dynamic responses with heterogenous latencies, may arise (Goldman, 2009; Harvey et al., 2012; Rajan et al., 2016). This may be through a highly structured feedforward architecture (Goldman, 2009), or a random network with minimally adjusted connections (Rajan et al., 2016). It is plausible that such architectures could also account for a heterogeneity of timescales. For instance, neurons at different positions in a feedforward network may have a different timescale as well as latency, although this has yet to be explored.

Recent work employing artificial spiking recurrent neural networks (RNN) has also provided further evidence of the importance of local connection patterns in determining neuronal timescales (Kim and Sejnowski, 2020). RNNs trained to perform working memory tasks were shown to contain neurons with a heterogeneity of timescales (Figure 5D). As in the electrophysiological data, neurons with longer timescales exhibited stronger and more stable encoding. Interestingly, a heterogeneity of timescales only emerged once RNNs had been trained to perform a temporally extended task (as opposed to a task not requiring the maintenance of information across time; Figure 5D), and was most dependent upon local connection strengths. Surprisingly, the connection strengths between pairs of inhibitory neurons were particularly important. Despite the fact that the networks were trained with a biologically implausible gradient descent learning algorithm, it will be important to explore these insights with more biophysically realistic network architectures along with experimental data.

### Inter-regional Connection Patterns and Cortical Layer

In addition to local connectivity, the other vital architectural feature facilitating heterogenous timescales across brain areas in a biophysical circuit model was the pattern of inter-regional connectivity (Chaudhuri et al., 2015; Figures 1D, 5B). The specific constellation of inputs and outputs to a brain region was critically important in determining its timescale. In short, the higher a brain region is located in an anatomical hierarchy, as defined by its inter-areal connections (Markov et al., 2014a), is strongly predictive of its average neuronal time-constants (reviewed in Wang, 2020). When applying this insight to single neurons, we should consider connectivity profiles at the level of cortical regions merely a helpful sketch of an infinitely more detailed neural architecture. Within a given brain region, the incoming and outgoing projections from each neuron are inevitably varied. A minority of neurons may receive direct projections from other regions, or be closely connected with other cells that do, whereas further neurons may be relatively distant from extra-regional input. Therefore, it is possible this heterogeneity in inter-areal projections may be another contributor to determining single neuron timescales.

The inter-region connectivity profile may relate to the cortical layer within which the neuron is situated. For instance, neurons with feedforward connections typically reside in supragranular layers, while those with feedback connections inhabit the infragranular layers (Felleman and Van Essen, 1991; Markov et al., 2014b). Interestingly, the cortical layer may also determine the degree of local connectivity, with neurons in layer III of prefrontal cortex thought to have particularly strong recurrent connections (Goldman-Rakic, 1995; Kritzer and Goldman-Rakic, 1995) reflected by an increase in spine density in prefrontal and parietal cortices relative to early sensory areas (Elston, 2003; Elston et al., 2011; Gilman et al., 2017). Recent studies have leveraged new technologies to demonstrate that task-related working memory activity mainly resides in supragranular layers (Markowitz et al., 2015; Bastos et al., 2018; Finn et al., 2019), providing experimental evidence that recurrent circuitry may be important for generating persistent activity. In future studies, laminar electrode probes may also provide insight into the relationship between neural timescales and cortical layer.

Beyond single neuron electrophysiology studies, recent work has shown that the functional connectivity between brain regions, as determined by resting state fMRI BOLD signal or magnetoencephalography (MEG), is also closely related to the hierarchical heterogeneity in local circuit properties (Demirtaş et al., 2019). A large-scale biophysical model of cortex, with the intrinsic properties such as the levels of excitation and inhibition of individual brain regions varied according to their hierarchical position (Burt et al., 2018), was able to closely mirror human resting state functional connectivity measures (Demirtaş et al., 2019). It was also able to predict a hierarchical topography of spectral features of resting-state MEG. An important advance of this study was that it accounted for heterogenous circuit properties between regions (although not within them). This suggests that at a more local level, the within-region heterogeneity we have discussed in this article (which we posited to be important in determining timescale) may also have an important influence on functional connectivity and oscillatory activity. This provides a link between neuronal timescales and large scale brain networks.

### Cell Type and Receptor Expression

The neuron type, for instance whether it is excitatory or inhibitory, likely has an impact on a cell’s timescale. In prominent spiking circuit models for extended cognitive processes, such as decision-making and working memory, pyramidal cells and interneurons play different functional roles (Brunel and Wang, 2001; Wang, 2002). Subgroups of pyramidal cells exhibit stimulus-specific persistent activity for particular choice options or memoranda, while interneurons provide non-selective inhibition. If this architecture is indeed present in primate cortex, it is likely excitatory neurons embedded within richly reverberant pools should have longer timescales than interneurons, as well as other non-selective pyramidal neurons. Some recent experimental evidence using neuronal spike width as a proxy for cell type suggests that the ratio of putative pyramidal to inhibitory neurons increases progressively up the cortical hierarchy, possibly facilitating stronger persistent dynamics (Torres-Gomez et al., 2020). Although using this technique could reveal information about the biophysical basis of single neuron timescales, a more reliable investigation of the role of different cell types may require experimental techniques currently only available in rodents. This may also uncover dissociable timescales in different types of GABAergic interneurons which are hypothesized to play distinctive roles in persistent activity (Wang et al., 2004).

Another important determinant of neuronal timescales may be neurotransmitter receptor expression. Slow decaying NMDA receptor (NMDA-R) synaptic currents, which allow post-synaptic neurons to remain depolarized for a greater length of time, are thought to be critical for the stability of neural activity (Wang, 2001). NMDA-R expression is variable across neurons, and given its importance for persistent activity, likely contributes to a neuron’s timescale. This could be tested empirically using iontophoresis of NMDA-R antagonists (Wang et al., 2013). The specific subunit combination of the NMDA-R may also be of relevance. NMDA-R are heterotetramers, meaning they are the assembly of four distinct subunits. Each NMDA-R typically consists of two NR1 subunits, together with two NR2 subunits. While the eight possible splice variants of the NR1 subunit are relatively similar, the four varieties of NR2 subunits (NR2A, NR2B, NR2C, NR2D) are more heterogeneous. The NR2 subunit expressed in each receptor is therefore important in determining its kinetic properties (Monyer et al., 1994; Vicini et al., 1998). The NR2 subunits are differentially expressed across different cell types, brain regions, and at different stages of development (Watanabe et al., 1992; Monyer et al., 1994). Interestingly, recent work has shown that the NR2B subunit is increasingly expressed further up the anatomical hierarchy, with the greatest expression in prefrontal cortex (Wang et al., 2008; Burt et al., 2018). Therefore, at a more local level, the degree to which a neuron expresses the NR2B subunit may be important in determining its timescale. In addition to the NMDA-R, other neurotransmitter systems may be relevant, particularly those exerting ascending neuromodulation such as dopamine (Arnsten et al., 2015) and acetylcholine (Croxson et al., 2011).

In summary, we have discussed a broad range of factors which may contribute to a neuron’s timescale—namely cellular morphology, connectivity profile, and receptor expression (Figure 5A). When randomly sampling neurons within the macaque prefrontal cortex, the morphology, cell-type, cortical layer and synaptic features are unknown. Recorded neurons are therefore likely sampled from separate subnetworks with differing underlying properties. This may explain the heterogeneity observed in neuronal timescales.

## Computational Advantages of A Diversity of Within-Region Neuronal Timescales

The general advantages of processing information across a range of temporal scales at the whole brain level are clear. Short timescales allow one to respond rapidly to important changes in the environment, while long timescales facilitate the integration of information to improve the signal-to-noise of working-memory and decision-making computations. Previous perspectives have addressed the computational advantages of a diversity of processing timescales in detail, and suggested these processes may occur in different brain areas. However, here we will specifically consider why it would be computationally advantageous for *individual brain regions* to also possess their own diversity of timescales.

While the distinction of neuronal timescales at the level of cortical regions has proven important, this has most commonly been framed in the context of processing simple sensory stimuli. In reality, the brain must also process much more complex features of the environment across a range of timescales. The computation of many of these complex features is limited to cortical association areas, as neural computations are constrained by a region’s anatomy. These computations often require the integration of many different attributes, but not necessarily across time. To compute the value of a reward—its probability, magnitude, any delay before receiving it, and the acquisition costs must be integrated. Like transient sensory stimuli, values can also evolve sporadically. It is thus important that values are dynamically tracked to facilitate rapid responses to the sudden appearance of a highly rewarding stimulus that is too good to miss, but also integrated gradually across time to improve signal-to-noise ratio and maximize decision-making accuracy. By extension, if the computation of complex features such as value are limited to higher cortical regions, it would be advantageous if neural populations *within* cortical association regions also had a range of diverse timescales for processing value. There is now some experimental evidence showing how this may occur—with neurons in cingulate cortex showing different responses according to their timescale. During an inter-trial period, short timescale neurons signalled the outcome from the immediately preceding trial, whereas long time scale neurons encoded a separate piece of information which was relevant to future decisions on subsequent trials (Fontanier et al., 2020).

Another useful implementation for this diversity of timescales would be in reinforcement learning (Sutton and Barto, 1998). Here, agents compute reward expectation by using a temporal filter to weigh previous outcomes. The optimal timescale for the filter is dependent upon the volatility of the environment; in a stable setting a long temporal filter allows more accurate predictions, whereas in a dynamic setting a short temporal filter should be employed to track changing payoffs (Behrens et al., 2007). Through applying a differential weighting to neurons with different reward timescales in response to changes in environment volatility, efficient reward expectations could be estimated. There is already experimental evidence for heterogenous reward timescales, with neurons integrating to different degrees across previous outcomes (Bernacchia et al., 2011). A similar concept has been explored in a recent neuroimaging study, where ACC was shown to possess a range of learning rates when humans made decisions in a volatile environment (Meder et al., 2017). It would be interesting for future studies to explore how these timescales are utilized. Specifically, whether the outputs of neurons with different timescales are indeed weighted differently by a decoder somewhere within the brain according to the current environmental volatility. This would be in line with similar previous observations of how neural population activity can be flexibly weighted according to current behavioral demands (Raposo et al., 2014), and shed light on how a brain region may utilize its diversity of timescales.

A brain region potentially capable of implementing these ideas is the ACC. ACC neurons not only encode choice and reward history (Seo and Lee, 2007), ACC activity encodes reward information and learning rates over diverse temporal scales (Bernacchia et al., 2011; Meder et al., 2017). Moreover, in the case of both the anatomical connection density patterns (Chaudhuri et al., 2015) and intrinsic neuronal time constants (Murray et al., 2014; Cavanagh et al., 2016), ACC is at the top of the cortical hierarchy, potentially organized in local anatomical gradients (Meder et al., 2017). The simultaneous representation of multiple time constants in ACC may allow the computation of reward trajectories by comparing estimates of recent and past reward rates.

In addition to adaptively weighting neurons according to their timescales, the temporal dimensionality of neural representations is also relevant for decoding. When encoding an item in working-memory, one computational perspective suggests that a stable pattern of neural population activity is preferable—as irrespective of the passage of time, a downstream decoder can utilize the same readout weights for the interpretation of a mnemonic representation (Murray et al., 2017). As we have shown earlier in this piece, neurons with long timescales may be particularly adapted to perform this stable maintenance function (Cavanagh et al., 2016, 2018). A recently emerging, and highly influential, concept in computational neuroscience has been the importance of mixed selectivity in maximizing the dimensionality of neural representations (Rigotti et al., 2013; Fusi et al., 2016; Stringer et al., 2019). In tasks with multiple features, prefrontal neurons generally encode these features with non-linear interactions, and this in turn maximizes the number of different available linear classifiers which could be utilized for readout. In addition to mixing the activity across neurons, varying the activity across time would further increase the dimensionality (Wasmuht et al., 2018). Therefore, while the stable coding schema offers some advantages, this is at the expense of minimizing the possible dimensionality of encoding relative to a population whose activity varies across time. By possessing subpopulations of neurons with different timescales, the prefrontal cortex is simultaneously providing easily-interpretable readout, as well as a high dimensional one—dependent upon which neurons a downstream decoder chooses to listen to at any given time. This would appear an important advantage of individual brain regions being capable of processing information across different timescales.

In addition to determining the value of stimuli, flexibly applying abstract rules is another important aspect of higher-level cognition (Miller and Cohen, 2001). It requires an agent to modify their response to a stimulus according to dynamically changing contexts or goals. Similar to value computations, experimental evidence suggests the neural substrates for rule based processing reside within higher cortical areas such as prefrontal cortex (Buckley et al., 2009); with neurons encoding abstract rules and rapidly altering how stimulus features are mapped onto actions (Wallis et al., 2001; Buschman et al., 2012; Mante et al., 2013). These rules are often implemented in a hierarchical fashion (Botvinick et al., 2009) which naturally necessitates the organization of behavior at a range of different timescales. Such behaviors often need to be applied rapidly based upon a single salient piece of information, and this would not be possible if prefrontal cortex was only capable of processing information across long timescales as suggested from previous studies (Murray et al., 2014; Hasson et al., 2015).

Another cognitive process which may involve a diversity of neuronal timescales is evidence accumulation. Evidence accumulation refers to the process by which information favoring alternative hypotheses is gradually integrated over time, and has been proposed to underlie perceptual, value-based, and many other forms of decision (Gold and Shadlen, 2007; Krajbich et al., 2010; Shadlen and Kiani, 2013). A series of recent behavioral studies have revealed that the timescale across which evidence is accumulated can be flexibly adjusted according to features of the stimulus or environment (Ossmy et al., 2013; Glaze et al., 2015; Bronfman et al., 2016; Levi et al., 2018; Piet et al., 2018; Ganupuru et al., 2019). For instance, in change detection tasks, humans weigh evidence differently according to how long they expect the intervening “change” in a noisy background stimulus to last (Ossmy et al., 2013). By adopting a shorter accumulation timescale for expected signals with a briefer duration, humans can perform this challenging task effectively. One mechanism by which the decision timescales could be adjusted is through individual brain regions having access to neural representations accumulating evidence across a diversity of timescales- as we have proposed in this review. This solution would provide a flexibility which could solve many more complex problems faced in the real world. For instance, Ossmy et al. (2013) contemplate a real-world example whereby a radar operator must interpret whether signal fluctuations may represent a missile, a passenger plane, or noise. In this problem, the brain must simultaneously accumulate evidence to detect the two important features (missile and plane), which may have different signal patterns/durations. A brain region utilizing a heterogeneity of timescales and applying them to integrate the same visual signal would be well suited to solve this problem. Another example of where this may be useful is situations where different types of decisions, which may use different criteria, must be made based on the same stimulus. A recent study suggested that during a similar change detection task, humans used separate timescales for the initial decision that they had detected a stimulus change, and a second decision to gauge their confidence (Ganupuru et al., 2019). This provides further evidence to suggest that the brain simultaneously has access to multiple neural representations of accumulated evidence across different timescales. These concepts will need to be explored further in future neurophysiological studies probing flexible timescales in evidence accumulation.

Interestingly, work from computational modeling studies suggests that a heterogeneity of timescales is not a default property of neural networks (Kim and Sejnowski, 2020). This heterogeneity only begins to emerge after the network is trained to perform a temporally extended task. Networks trained to perform a simpler response-based task, without any temporal component, had shorter and less heterogenous timescales. This is further evidence that this heterogeneity is present to support the computations discussed in this review: decision-making and working-memory.

Although many of the studies above focus on the processing of task-relevant stimuli, it is also likely that a similarly broad range of timescales of operation may be needed when performing motor control; in particular for temporally extended sequences of complex actions. For example, a recent theoretical account of motor cortex dynamics used a network model with balanced excitation and inhibition to generate “stability-optimized circuits” (SOCs) that could generate complex movements (Hennequin et al., 2014). The authors found that in order to generate such movements, the time constant of membrane and synaptic dynamics in the SOCs (~200 ms) had to be set to match the dominant timescale in the data they were trying to model (Churchland et al., 2012), giving these connections a slower time-constant than other randomly connected synapses in the same model. They argued that such segregation of fast and slow time-constants may even arise within the same neuron, *via* the respective contribution of proximal and distal synapses in the dendritic arbor. Further evidence supporting a diversity of timescales within a single motor control region comes from functional MRI studies of sequential skilled motor performance in humans (Yokoi and Diedrichsen, 2019). Here, contrary to the common hypothesis of an anatomical division of labor between different levels of a motor control hierarchy, it was instead found that the representation of (short-timescale) movement “chunks” and (long-timescale) movement “sequences” can be spatially overlapping in premotor and parietal areas.

One important consideration is how the resting autocorrelation time constants of individual neurons (generally ranging from tens to hundreds of milliseconds) can be related with behaviors that occur across timescales which are orders of magnitude longer. Many of the behaviors discussed in this section occur across timescales much greater than the longest individual neuronal timescale measured. This likely reflects that these behaviors are generated by network-level states, to which the contribution of individual neurons is at least somewhat redundant. Furthermore, as the timescales are assigned during a short window of resting activity, their values likely reflect only a fraction of the duration of persistent activity which could potentially be supported. It is also important to remember this same challenge applies to current circuit models which have assigned timescales of similar magnitudes to cortical regions (Chaudhuri et al., 2015).

## Implications for Future Electrophysiology Studies

In this review, we have demonstrated that an individual neuron’s intrinsic timescale while at rest provides insight into its functional properties and roles during cognitive tasks. This has important implications for how neurophysiological datasets are collected and analyzed. For instance, one commonly employed tactic in neurophysiology recordings in areas such as the lateral intraparietal sulcus has been to preselect which neurons to record from based upon their properties during a memory-guided saccade task (Gnadt and Andersen, 1988), in order to establish that neuron’s receptive field. When using this technique, investigators select neurons which exhibit stable, persistent activity before examining their properties during a cognitive task of interest. It is therefore likely that they are predominantly sampling neurons with longer timescales. While this approach has proven fruitful, and it is understandable given the technological challenges of recording from sufficient numbers of neurons, it has likely led to a biased perspective of the overall neural dynamics. For instance, it may have overstated the proportion of neurons exhibiting stable activity during working-memory tasks and gradual ramping activity during perceptual decision-making (Goldman-Rakic, 1995; Gold and Shadlen, 2007). This is important because it entirely overlooks the roles of neurons with non-classical response profiles. It also has arguably led to an over-emphasis of the capabilities of individual neurons, supported by idealised examples, and the disregarding of more sophisticated population-level solutions to computational problems (Rigotti et al., 2013). A more complete understanding of neural computations requires us to understand the roles of all of the neurons in these cognitive processes, and the recording of as representative a sample as possible in order to appreciate how neurons function together as a population. Fortunately, new technologies are becoming increasingly available that will allow investigators to record from many neurons simultaneously across each cell layer (Sofroniew et al., 2016; Jun et al., 2017). This should hopefully facilitate a more unbiased characterization of the heterogeneity of neuronal responses. If researchers are particularly interested in a certain subpopulation of neurons with stable activity, they will still be able to find these neurons *post hoc* using the timescale method discussed in this review. However, they will also have access to a plethora of extra information about what neurons with other timescales are doing, and how the population as a whole behaves.

Although this review has primarily focussed on macaque neurophysiology studies, future work may also seek to apply the timescale analyses to high-density electrophysiological recordings collected from rodents (Siegle et al., 2019); where cognitive processes are being studied with an increasingly sophisticated repertoire of techniques. These include the precise perturbation of neural circuits, recording from genetically identifiable neurons and the implementation of neuropsychiatric disease models. These experiments would provide some more concrete insights into the determinants of single neuron timescales.

One important limitation of the majority of neurophysiological datasets considered in this review is that they study a behavior which requires the prolonged maintenance/integration of information. It therefore makes sense that a prominent role for long timescale neurons in these computations was established. However, to fully explore the functional importance of a diversity of timescales, tasks which require tracking information (and modulating behavior) over both short and long timescales should be explored (Behrens et al., 2007; Daw et al., 2011; Massi et al., 2018). Future work should try to establish if there is an important role for neurons with shorter timescales in such tasks. Another important consideration is to study a task which simultaneously requires the dynamic tracking of complex information, as well as its gradual integration. As suggested earlier in this piece, one possibility would be to record neurophysiological activity on an evidence integration task, where subjects must combine many samples with unique characteristics. This would make clearly dissociable predictions for the neural representations to expect in shorter timescale (momentary evidence) and longer timescale (integrated evidence) neurons.

Furthermore, while the partition of neurons into short and long timescale provides intuition and is necessary when analyzing the patterns of coding at the population level, it is a relatively coarse simplification of the underlying concept of a heterogeneity of single-neuron timescales. Ideally, a task design would demonstrate the utility of a diverse continuum of timescales. For instance, subjects could be trained to temporally filter previous rewards across a different number of trials according to a cue presented each trial. This should require the processing of reward across a range of timescales, and the trial-wise adaptive weighting of each timescale population dependent on the behavioral cue.

## Conclusion

In summary, we have reviewed important electrophysiological evidence from a series of recent studies that convincingly demonstrate the heterogeneity of timescales at the level of single neurons within a cortical region. This heterogeneity is functionally relevant for the computations that neurons perform during decision-making and working memory. A neuron’s timescale is likely determined by the neurotransmitter it releases, its local connectivity pattern, receptor expression, and cortical layer.

It is important for individual brain regions to have neurons with a heterogeneity of timescales, as many high-level cognitive processes such as learning, planning, and rule-based behavior require making adaptive decisions to changing environmental demands. These computations generally occur in higher cortical regions which have a long timescale when considered as a whole-brain region, but individual neurons in these areas display a diversity of timescales. A heterogeneity of timescales also offers a compromise between robust stable representations that are easy to read out and those which are most efficient and high dimensional. Future experimental work further demonstrating some of the advantages of short timescale neurons in higher cortical areas, and how a population may effectively utilize a whole distribution of timescales, will further strengthen our arguments about their computational role. The method we have outlined has already provided important computational insights and will prove an increasingly valuable tool as researchers start to record from more neurons simultaneously.

## Author Contributions

SC: conceptualization, writing—original draft, writing—review and editing. LH and SK: conceptualization, supervision, funding acquisition, writing—review and editing. All authors contributed to the article and approved the submitted version.

## Conflict of Interest

The authors declare that the research was conducted in the absence of any commercial or financial relationships that could be construed as a potential conflict of interest.
